# Individuality really matters for fish welfare

**DOI:** 10.1080/01652176.2023.2270653

**Published:** 2023-10-16

**Authors:** Caroline Marques Maia

**Affiliations:** a FishEthoGroup (FEG) Association, Faro, Portugal; b Alianima, São Paulo, Brazil

## Introduction

Animal welfare science started to gain ground in the 1960s (Duncan 2006), especially after the publication of the famous book Animal Machines in 1964 by Ruth Harrison. This book exposed several farm practices that were causing suffering for terrestrial farmed animals, which later basically culminated in the official publication of the famous five freedoms in 1979 by the Farm Animal Welfare Council. Since then, the animal welfare concept has evolved over the years. Nowadays, there is a tremendous amount of data and literature on many different issues relating to animal welfare, including the idea that not only avoiding negative aspects are important, but also adding positive stimuli in the environment to improve the captive conditions of animals is important (Mellor and Beausoleil 2015). In the process of animal welfare development through the years, concerns have reached many other domains involving human-animal interactions and relationships, like laboratories, zoos, domestic environments, labor, and conditions related to human leisure, thus reaching fishes.

Over the years, quite some progress has been made on the issue of considering, understanding, and measuring fish welfare in many different species, which is clearly indicated by a raising number of books covering a wide range of related topics (e.g. Branson 2008, Kiessling et al. 2012; Arechavala-López and Saraiva 2019; Kristiansen et al. 2020; Studer 2020). Furthermore, there are already important projects on assessing the welfare conditions in farmed fishes, such as the fair-fish database or the English version of the FISHWELL Atlantic salmon welfare handbook (Noble et al. 2018). Models for welfare assessement of fishes have been proposed (e.g. Pettersen et al. 2014) and fish welfare guidelines or reports are also becoming more common (e.g. Saraiva et al. 2021; Arechavala-López 2022; Saraiva 2022).

Despite that, fishes are still commonly neglected animals in practical terms of welfare. This fact is even more relevant when it is taken into account that such aquatic animals are present in farms, laboratories, fisheries, public aquariums, home aquariums and recreational fishing. In this scenario, it is also worth mentioning that, compared to other animals involved in relationships with humans, there is a countless number of fish species that is used by humans in one way or another. Furthermore, it is important to consider that some of these species are more domesticated than others, each one with its own natural behavioural needs and preferences, which also depends on the life stage of the species.

## Fishes are sentient animals expressing individual responses

### Sentience

The issue about sentience in fishes is fundamental to fish welfare concept and its moral significance. Sentience refers to the capacity of an animal to experience basic emotions, in particular discomfort and pain, then related to its capacity of suffering. The question about the possibility of fishes feeling pain have been the reason for intense discussions in the field of animal behaviour and welfare (e.g. Vettese et al. 2020; Debating Fish Pain Forum). Because the central nervous system of fishes is simpler than mammals and birds, some authors argued that such animals are not capable of experiencing pain as they lack the neocortex, or any functional equivalent (e.g. Rose 2002; Rose et al. 2014). Despite that, studies have demonstrated over the years that, as mammals, fishes have nociceptors receiving painful stimuli and nerve fibers that conduct such painful information to their brain (Sneddon et al. 2003a; Dunlop and Laming 2005; Braithwaite and Boulcott 2007, Sneddon 2015), as well as where this information is processed (Dunlop and Laming 2005; Braithwaite and Boulcott 2007; Nordgreen et al. 2007; Sneddon 2015). In fact, there are evolutionary conserved features in fish brain, as well as newly acquired ones, like in the developing and adult zebrafish thalamus, for instance, compared to the mammalian situation (Mueller 2012). Zebrafish is even considered a powerful model for studying human inherited neurological conditions, both in terms of delineating underlying mechanisms and developing therapeutic strategies (Kozol et al. 2016).

Moreover, fishes express complex behavioural alterations when feeling pain (Sneddon et al. 2003a; 2003b; Braithwaite and Boulcott 2007; Sneddon 2015), which are significantly minimized if they receive analgesics (Sneddon et al. 2003b; Sneddon 2015). For instance, painful events result in reduced activity, impaired guarding behaviour, suspension of normal behaviour, increased ventilation rate and abnormal behaviours in fishes, which may be all prevented by the use of pain-relieving drugs (for review, see Sneddon 2019). Additionally, zebrafish was already demonstrated to choose receiving analgesics if it has the choice when exposed to a painful condition (Sneddon 2013). In fact, zebrafish models can be considered as emergent tools to explore pain behaviors, pain-related mood disorders, and to facilitate analgesic therapy screening in translational pain research (Costa et al. 2022). Furthermore, fishes may express behavioural changes indicating other negative affective states, such as anxiety and fear (for review, see Braithwaite and Boulcott 2007; Maximino et al. 2010). Such aquatic animals are also able to show incredible cognitive abilities, such as nest construction (Fryer and Iles 1972), which can be used for a variety of different functions beyond spawning and parental care (for review, see Bessa et al. 2022). They are also capable of tool use (Brown 2012), storing long term memory (Csányi et al. 1989; Triki and Bshary 2020), and recognizing themselves (Kohda et al. 2023) or even human faces (Newport et al. 2018).

The consensus amongst scientists increasingly indicates that fishes can suffer, although much is still unknown about what exactly makes them suffer or what their preferences are in several different circunstances, then creating the need to deal with uncertainty in many cases (Bovenkerk and Meijboom 2013). There are so many different species and such great variability between them, that research done in one species does not unproblematically translate to another species (Bovenkerk and Meijboom 2013), and this should be taken into account in a practical way. In fact, a huge variation in emotional/cognitive systems of fishes and the underlying neuroanatomy may exist between different species, especially given their vast number (about 30.000 species), which live under several different ecological conditions (Kristiansen et al. 2020). Despite that, as proposed by Bovenkerk and Meijboom (2013), in the absence of absolute certainty, if we still have good reasons to believe that certain measures improve fish welfare, we should apply them. Taking this into account, it is already past time to clearly deal with another characteristic found in fishes that has a great impact in their welfare conditions: fishes express significant individual responses even within the same species.

### Individual physiological and behavioural differences

The authors of the paper *Looking beyond the Shoal: Fish Welfare as an Individual Attribute*, which was published at Animals Journal last year (Torgerson-White and Sánchez-Suárez 2022), reminded us that fishes express considerable individual variation in cognitive abilities, emotions, and preferences, which are linked to personality (Budaev and Brown 2011). Despite similar topics were already highlighted before, like in a whole special issue dedicated to the relevance of fish individuality when assessing their physiology, welfare and performance (Gesto et al. 2020), it is still not widely recognized. Different personalities or behavioural traits refer to some individuals coping differently from others with the challenges that the environment poses on them. In fact, this issue has been addressed in fish species in several different ways over the years (e.g. see the chapter of Johansen and colleagues in the book The Welfare of Fish, Kristiansen et al. 2020). For instance, thresholds for employing active (proactive) and passive (reactive) responses under stressful situations are individually variable, and complex gene-environment interactions affects the occurrence and stability of welfare relevant trait correlations in these cases (Johansen et al. 2020). Therefore, key components of a stress coping style are subject to great individual and heritable variation, and such specific trait characteristics may directly influence the welfare of fishes (Johansen et al. 2020).

In this scenario, considering conditions that fishes are kept under high stocking densities, one important question that remains is related to how different behavioural traits or personalities may function within a social group or a shoal. Even under the most optimal conditions, it is clear that not all individuals cope similarly, as many studies have shown. On the other hand, removing individuals that seem to be suffering under the imposed conditions from stable social groups is risky, as it may also lead to problems with aggressiveness and hierarchical relationships. Therefore, assuring individual welfare for fishes when living socially is a big challenge, especially at high stocking densities.

## Dealing with fish individuality

### How to measure and to assure individual fish welfare under high densities?

Since we consider animal welfare as an individual attribute and know that fishes, in fact, express consistent individual differences within the same species, it is hard to imagine how it would be possible to assure better welfare conditions for all these animals when they are kept under high densities. Under high stocking conditions, fish welfare assessment frequently involves sampling individuals from rearing units followed by evaluating their physiological, nutritional, anatomical and even behavioural indicators individually. Thus, the common procedure normally involves using some fishes as representatives of the rearing units, which, of course, does not allow to easily finding specific individuals that might be suffering under poor conditions regarding their own individual needs. Whereas this kind of approach is helpful to assess fish welfare in general, it means that individual welfare needs is overlooked. To make matters worse, this is clearly the case for farm and some lab conditions, like holding tanks. As pointed out in by Torgerson-White and Sánchez-Suárez (2022), it is much easier to deal with this issue in conditions where fishes are kept under low densities, because this could be comparable to improving the welfare of animals in zoos or under some lab conditions, with just a few fishes per tank, for example.

The issue of how to measure and assure the welfare of individual animals kept in large numbers like in farms is not unique to fishes, as the same applies for other farmed animals. By trying to propose some possible ways to deal with this issue once it is understood, Torgerson-White and Sánchez-Suárez (2022) made some suggestions in their paper. The authors proposed that more technology, such as video-monitoring, should be used to follow fish behaviours individually, aiming to better evaluate the welfare conditions of farmed fishes in aquaculture systems. This approach could help to early detecting anomalous behaviours and fishes in poor nutritional condition or even damaged. Despite the fact that this is not applicable in every case due to socioeconomic reasons, it is an interesting approach to help addressing individual needs and preferences under high stocking conditions. Another proposition from these authors is to investigate the individual variation of welfare state in grouped fishes, in a way that individuals are kept at low densities, which still enables to evaluate the welfare conditions individually. This should probably be conducted under more controlled conditions in laboratories. Such studies, or even research conducted in simulated farming conditions, may give hints about what is better for grouped animals in farms. It is much easier to monitor behaviours, physiological patterns, and preference responses of a few individuals in a tank than at a high density condition. In fact, based on studies with several individuals, criteria have been defined for salmons, assuring that most individuals will cope well in sea pen system (Pettersen et al. 2014).

Related to this point, Torgerson-White and Sánchez-Suárez (2022) highlight that natural behaviours and responses of fishes expressed in isolated conditions may vary when they are grouped. Thus, it makes sense that evaluating individual welfare of fishes within groups rather than in isolated individuals is important for better results that are applicable in captive conditions involving high densities, like farms. However, this does not seem to be an easy task, because it is also possible that individual responses vary between different group formations. Thus, despite the fact that such approach clearly represent an important step to better deal with individual fish welfare under high stocking densities, we should keep in mind that they always need validation under real farming conditions. Social context is of great impact on individual behaviours, and what is investigated in terms of welfare in low-density conditions is very often not applicable when the fish is in a different social condition, such as aquaculture-like densities. In fact, current literature is generally missing studies considering fish welfare in situations resembling actual farming conditions, or experiments performed directly on-farm.

Together, these arguments raise the question about how many fishes are still suffering when trying to safeguard the welfare of most individuals, and then how much suffering may go unnoticed using this kind of approach, as individual behavioural responses may vary between different stocking densities and it is not possible to follow all individuals over time. Thus, there is a great risk of missing suffering of individuals at high stocking densities, yet these kinds of aquaculture systems are economically viable and profitable. Therefore, working to better assure the welfare conditions of as most individuals as possible remains as the best option, because even considering that not all individuals are attended, at least most of them are potentially covered in their needs and preferences. Thus, investing in methods of measuring the welfare of most individuals or, at least, a representative number of them, is a good option to better reach this goal.

### Raising awareness about fish individuality

An important step to increase awareness about the importance of considering individual responses of fishes is to start recognizing that the animals inside a tank, aquarium, pond, cage or any other captive system are individuals *per se*. Therefore, the densities in which fishes are maintained should be given as ‘individuals’ rather than ‘kilograms’ per liter, cubic meter or gallon, as can still be found in some papers. In this line, fish yield in a production system should be mentioned in terms of the total number of individuals instead of just reporting the final kilograms or tons reached in such system. As highlighted by Torgerson-White and Sánchez-Suárez (2022), even the Food and Agriculture Organization of the United Nations (FAO) still reports aquaculture production in terms of tons rather than also indicating individual fishes. In this line, a proposition that could help to start seeing fishes as individuals was suggested by Jonathan Balcombe (2016). This author proposed to replace the word *fish* by *fishes* in the English language used in papers and other scientific communication or dissemination materials to identify two or more individuals of a same species, rather than using the word *fishes* just when more than one fish species is considered.

Furthermore, it is always important to have in mind and to clarify that contrary to many other animal groups that are under human caring, like chickens, broilers, cattle, pigs, cats, dogs, horses, etc, fishes are in fact composed by thousands of different species, with their own behavioural needs and particular characteristics, as already pointed out above. Hundreds of them are farmed worldwide and certainly much more than that are considered as ‘ornamental’ fishes, which is not a good word to define pet fishes, because ‘ornamental’ gives the wrong idea that fishes may be treated as mere objects. Thus, showing that there are several different species expressing clear individuality, even among individuals of a same species, is an important step to highlight that individuality of fishes matter for their welfare considerations.

## What should we do?

Even considering it will be a challenge to practically deal with the individual variability of responses in fishes, this must not prevent the search for reliable and applicable ways to face that. As briefly argued above, based on ample scientific evidence, fishes are able to suffer and express significant individual variation of different responses, which then must be taken into account when trying to improve their welfare conditions. In situations where they are kept at low densities, like home aquariums or some laboratory conditions, it is crucial that such individuality is considered when trying to improve the welfare of such individuals. Therefore, investigating more the individuality of fishes in aquariums or tanks, and providing them with tailor-made solutions by focusing on their individual preferences and needs is fundamental to improve their welfare conditions.

However, although it is clear that accounting for fish individuality in farms is needed, we should not forget that the main problem for the quality of life for fishes under farming conditions is that, in general, welfare considerations is commonly not considered at all, regardless if taking into account individual responses of fishes or not. Proper regulations about fish welfare under farming conditions are only in place in a very limited number of countries and, in fact, reliable information about fish welfare status in aquaculture from papers, manuals, chapters, reports, guides and other technical-scientific sources is still very limited. On one hand, there is a lot of scientific information on how to address fish welfare, but, on the other hand, there is very little efforts on using this information to produce and share welfare data that may be used to assess, and then to improve the welfare of captive fishes in farms.

Therefore, under conditions in which fishes are grouped at high densities, such as in farms, focusing on optimizing farming conditions in ways that are demonstrated to improve fish welfare, at least of most individuals, is already a good start. This may be done, for example, by optimizing water quality, improving the design of rearing units considering their colour, shape, dimensions, bottom, light intensity, etc, and by providing environmental enrichment with heterogeneous units, thus in a way that fishes may have the chance to choose according to their own individual preferences. Moreover, trying to monitor individuals under water using new technologies and validating laboratory research to farming conditions are fundamental approaches to help better understand and address individual needs and preferences wherever is possible under high stocking densities. More studies evaluating individual responses of grouped fishes in situations as similar as possible with farming conditions seems to be an interesting approach urgently needed, which can highlight important future findings in this field. Additionally, it is important to mention that in cases fishes are farmed focusing on restocking of their natural populations, focusing on conditions that promote their future individual welfare and survival after release should also be considered.

Finally, we should also take into account that because hundreds of species are cultured for meat, scientific experiments or human leisure, important scientific research about fish welfare in general is still scarce for many of them. Thus, much more research in this field is still needed. In this line, future studies should also better investigate the proposed approaches discussed here, as well as other possible ways to address individual responses of fishes grouped at high densities to improve their welfare. Whereas the importance of considering individuality for fish welfare is not fully recognized, the publication of papers as the recent *Looking beyond the Shoal: Fish Welfare as an Individual Attribute* highlights an important issue that should be really taken into account. We must look beyond the shoal whenever is possible and, when it is not, we should look for new ways of doing that in the future, because individuality really matters for fish welfare.
